# *Aeromonas* and *mcr*–3: A Critical Juncture for Transferable Polymyxin Resistance in Gram-Negative Bacteria

**DOI:** 10.3390/pathogens13110921

**Published:** 2024-10-22

**Authors:** Nathan L. McDonald, David W. Wareham, David C. Bean

**Affiliations:** 1 Microbiology Research Group, Institute of Innovation, Science and Sustainability, Federation University Australia, Mount Helen Campus, P.O. Box 663, Ballarat, VIC 3353, Australia; nathanmcdonald903@gmail.com; 2 Blizard Institute, Queen Mary University of London, London E1 2AT, UK; d.w.wareham@qmul.ac.uk

**Keywords:** *Aeromonas*, *mcr*–3, *mcr*–3-related genes, polymyxins, colistin

## Abstract

Polymyxin antibiotics B and colistin are considered drugs of last resort for the treatment of multi-drug and carbapenem-resistant Gram-negative bacteria. With the emergence and dissemination of multi-drug resistance, monitoring the use and resistance to polymyxins imparted by mobilised colistin resistance genes (*mcr*) is becoming increasingly important. The *Aeromonas* genus is widely disseminated throughout the environment and serves as a reservoir of *mcr*–3, posing a significant risk for the spread of resistance to polymyxins. Recent phylogenetic studies and the identification of insertion elements associated with *mcr*–3 support the notion that *Aeromonas* spp. may be the evolutionary origin of the resistance gene. Furthermore, *mcr*–3-related genes have been shown to impart resistance in naïve *E. coli* and can increase the polymyxin MIC by up to 64-fold (with an MIC of 64 mg/L) in members of *Aeromonas* spp. This review will describe the genetic background of the *mcr* gene, the epidemiology of *mcr*-positive isolates, and the relationship between intrinsic and transferable *mcr* resistance genes, focusing on *mcr*–3 and *mcr*–3-related genes.

## 1. Introduction

Infectious diseases are among the leading causes of death globally. Emerging and re-emerging bacterial infections are a substantial burden on veterinary and human healthcare. Treating these infections is complicated by the emergence and spread of antimicrobial resistance (AMR). AMR has emerged due to the over or misuse of antibiotics in the treatment of human and animal diseases [1]. The current estimate of global mortality due to AMR bacterial infections is >700,000 people annually [2,3].

The spread of genes imparting AMR quickly results in organisms becoming resistant to multiple groups of drugs. The emergence of multi-drug resistance to recent antibiotics has necessitated the reintroduction of older classes of antibiotics, such as polymyxins, to treat infections by Gram-negative bacteria (GNB).

Polymyxins B and E (colistin) are polycationic peptides that serve as drugs of last resort for the treatment of multi-drug resistant GNB. Their mode of action is to bind to the lipopolysaccharide (LPS) of susceptible bacteria. This alters the permeability of the outer and inner membranes to Ca^2+^ and Mg^2+^ ions, resulting in the loss of the osmotic barrier, which leads to cell lysis [4,5]. Polymyxin B and colistin have been categorised as critically important antibiotics in human and veterinary medicine by the World Health Organisation due to the emergence of multi-drug resistant GNB [6,7,8].

In 2015, the first case of transferable colistin resistance was recorded in resistant strains of *Escherichia coli* and *Klebsiella pneumoniae* isolated from pigs in China. The gene responsible for polymyxin resistance was identified as the plasmid-borne mobilised colistin resistance (*mcr*) gene, *mcr*–1 [9]. Since 2015, an additional nine *mcr* gene families have been identified (*mcr*–2–10). The *mcr* genes encode phosphoethanolamine lipid A transferase enzymes (PET–A), which reduce polymyxin binding affinity by increasing the positive charge of the bacterial lipopolysaccharide membrane through the addition of a phosphoethanolamine transferase (PE) group [5].

Plasmid-borne polymyxin resistance in GNB jeopardises the drugs’ role as an “antibiotic of last resort” and represents an increasing threat to global public health [7,8]. While polymyxin resistance had been previously recorded in normally susceptible organisms, these instances typically resulted from chromosomal mutations, with no recorded cases of resistance imparted via horizontal gene transfer [8,9,10].

*Aeromonas* is a genus of GNB known to exhibit intrinsic resistance to polymyxins. Evidence suggests that the genes that impart polymyxin resistance in *Aeromonas* species may have “escaped” and become mobilised in other Gram-negative bacteria, including *E. coli*, *Salmonella*, and *Klebsiella*. The current review summarises our current understanding of polymyxin resistance and explores the evidence that suggests the globally disseminated *mcr–3* gene originated in *Aeromonas.*

## 2. Polymyxins and Resistance: The Antibiotics of Last Resort

Polymyxins are a class of naturally occurring lipopeptide antibiotics originally derived from the Gram-positive bacterium *Paenibacillus polymyxa*, from which their name is derived [11]. The cationic antibiotics were discovered in 1947 by Stansly [12] and Ainsworth and Brown [13] independently of each other. There are six polymyxin subtypes (A, B, C, D, E, F) which were all derived from *P. polymyxa*. Soon after their discovery, reversible nephrotoxicity and neurotoxicity were reported for all polymyxins in human cell lines [13,14]. Polymyxin B and colistin were determined to be the least toxic based on in vivo models and are the only types approved for clinical use. All polymyxins show similar levels of antibacterial activity [15]. Polymyxin B and colistin were introduced in clinical and veterinary settings in 1965 to treat GNB infections but were later removed from circulation and replaced by aminoglycosides in the 1980s due to emerging safety concerns regarding their neuro- and nephrotoxicity [14,16]. They were reintroduced in 1995 as drugs of last resort due to the emergence of multi-drug resistant GNB, predominantly to treat carbapenem-resistant organisms [8].

The mechanism behind the antibacterial activity of polymyxins is the electrostatic attraction between the cationic head of polymyxins and the anionic phosphate groups on lipid A of the LPS in the bacterial outer membrane [5]. The binding of these groups displaces membrane-bound divalent cations (Ca^2+^ and Mg^2+^) that stabilise the membrane [4]. The disruption destabilises the outer membrane, increasing its permeability and allowing the polymyxin’s hydrophobic tail to integrate into the lipid bilayer of the outer membrane [4,5]. The destabilisation of the outer membrane enables the polymyxin to cross into the periplasmic space and bind to the inner membrane [17]. Once bound, the polymyxins insert their hydrophobic tails into the inner lipid bilayer, creating non-selective pores within the membrane that cause the leakage of cellular constituents and result in cell lysis [17,18,19].

Prior to the discovery of *mcr*–1, it was believed that polymyxin resistance only developed through mutations in chromosomal components of the regulatory system (*pmrAB* and *phoPQ*), which modify PE and 4–amino–4–deoxy–l–arabinose (l–Ara4N), respectively [20]. Mutations in these genes result in the up-regulation of the *pmrCAB* and *arnBCADTEF*–*pmrE* operons, which encode the synthesis of Ara4n and PE [21]. Increased incorporation of l–Ara4N and PE into the LPS of GNB reduces the electrostatic interactions between polymyxins and the phosphate groups in lipid A and the inner core of the LPS. Lipid A is the lipid component of the endotoxin that anchors the LPS of GNB and constitutes the innermost region of the LPS. Its hydrophobic properties anchor it to the outer bacterial membrane and the hydrophilic oligosaccharide membrane core [20]. Unlike l–Ara4N modifications, which are initiated by chromosomally encoded enzymes, the addition of the PE group to lipid A is encoded by the phosphoethanolamine lipid A transferase gene (*PET–A*). This is present in the genomes of intrinsically resistant GNB and can be spread via horizontal gene transfer to susceptible organisms.

Our understanding of polymyxin resistance was set to change drastically in 2015 when the first case of transferable resistance was discovered in normally susceptible organisms, such as commensal strains of *E. coli* and *K. pneumoniae* isolated from pig farms in China [22]. The discovery of resistance imparted by the transferable *mcr*–1 gene was significant, as prior to this, the consensus was that resistance could only be chromosomally derived [23].

## 3. *mcr* Gene Variants and Resistance

Resistance to polymyxins is intrinsic in several genera of GNB, including *Neisseria, Serratia*, *Brucella*, and *Aeromonas* spp. Other GNB, including pathogenic *Enterobacteriaceae*, can become resistant via the accumulation of chromosomal mutations that modify their LPS cell membrane surface or by acquiring the *mcr* gene via horizontal gene transfer [10]. Following the discovery of the first *mcr* family *(mcr*–1) in 2015, an additional nine families have been identified in both humans and animals. The nomenclature of *mcr* genes consists of the gene family and the specific allele within that family, separated by a decimal point (i.e., *mcr*–3.1 is the first allele of the *mcr*–3 family). Four gene families were discovered in Europe (*mcr*–2, 4, 5, and 6), five in China (*mcr*–1, 3, 7, 8, and 10), and one in the USA (*mcr*–9)[24,25,26] (Table 1).

The mechanism of resistance imparted by *mcr* genes was determined by Hinchliffe and Yang [32] and shown to occur via the action of PET–A, similar to the mechanism involving l–Ara4N and PE modifications resulting from chromosomal mutations [32]. PET–A increases the positive charge of the LPS membrane through the addition of a PE group to anionic lipid A head groups. The transfer of PE from lipid A reduces its positive charge, reducing the binding affinity of polymyxin to the LPS [33]. PET–A is an intra-membrane enzyme whose structure consists of five hydrophobic transmembrane helices localised within the cellular membrane. Its structure consists of two folded domains connected by a bridged helix, a C–terminal periplasmic catalytic domain, and an N–terminal transmembrane domain [34]. Multiple conserved and partially conserved amino acid sequences have been suggested to be involved in the binding of PE groups; however, the lipid binding pocket remains poorly defined [30,32].

GNB with intrinsic resistance to polymyxins possess one or more *ipet–A* genes, which encode intrinsic lipid A phosphoethanolamine transferase (IPET–A) enzymes. IPET–A and its variants are also referred to as non-mobile *mcr* (NMCR) [30,35,36,37,38,39,40]. The *mcr*–encoded PET–A shares high sequence and structural similarities with IPET–A enzymes present in members of GNB that are intrinsically resistant to polymyxins, including members of *Aeromonas* spp. For example, *mcr*–1 shares 40% similarity in structure with PET–A [6].

## 4. *PET–A* and *mcr*: Some *mcr* Alleles Are More Closely Related to *PET*–A than Others

A wide range of genetic diversity has been observed within the nucleotide sequences of the *mcr* gene families and their variants [6]. Gaballa and colleagues [5] investigated *mcr* diversity in the context of intrinsic lipid modification resulting from *ipet–A* using an in silico model based on 69,814 bacterial genomes from NCBI BLAST. They determined that consistent with the wide range of nucleotide sequences, the amino acid sequence diversity among *mcr*-encoded PET–A varied widely, with sequence similarities ranging from 59.3% to 100.0%. Amino acid and nucleotide sequences associated with 237 chromosomally encoded IPET–A, IPET–B, and IPET–C (which modify lipids B and C in the LPS) were extracted from 147 genera that harbour *mcr,* including *Aeromonas*, *Citrobacter*, *Enterobacter*, *Escherichia*, *Klebsiella*, *Moraxella*, *Proteus,* and *Salmonella* spp. The analysis identified 98 known *mcr*-encoded PET–A and 125 novel PET–A variants. The *mcr* alleles displayed a low degree of similarity with IPET–B and IPET–C, ranging from 46.0% to 58.0% and 42.0% and 49.0%, respectively. The *mcr*-encoded PET–A allele’s similarity to *ipet–A* ranged from 61.0% to 76.0%, with some *mcr* alleles having higher similarity to *ipet–A* than others. Gaballa and colleagues concluded that sequence similarity cannot be used conclusively to differentiate *mcr*-encoded PET–A from *ipet–A*-encoded PET–A. A combination of phenotypic and molecular identification methods is required to identify *mcr*-positive isolates.

## 5. The *mcr*–3 Gene: An Overview

The first identification of *mcr*–3 was in *E. coli* isolated from pigs in China [10]. It was a 1626 bp gene that encoded *PET–A,* which shared a 45.0% and 47.0% nucleotide sequence identity and a 32.5% and 31.7% amino acid sequence similarity to *mcr*–1 and *mcr*–2, respectively. Using RaptorX protein structure prediction software (v2), Yin and colleagues predicted that the PET–A encoded by *mcr*–3, similar to *mcr*–1 and 2, has a structure consisting of two domains. The first domain contained five transmembrane alpha helices, and the second was predicted to be a periplasmic domain that contains its catalytic centre, which aligned with the domains of PET–A as identified by Anandan and colleagues. Wang and colleagues [41] determined that *mcr*–3 had the same level of PET–A enzyme activity as *mcr*–1 and 2, and that the expression of *mcr*–3 increased the MIC of colistin from 8 to 32 µg/mL. An analysis of the LPS via mass spectrometry by Kieffer [38] found that both *mcr*–1 and *mcr*–3 produced identical peaks at *m/z* 1921 in *mcr*–1- and *mcr*–3-positive *E. coli* isolates, resulting from the addition of PE groups. This demonstrated that differences in amino acid sequence did not necessarily impact PET–A functionality, suggesting that *mcr*–3 provides polymyxin resistance via the same mechanism as *mcr*–1.

The first *mcr*–3 genes were localised to IncHI_2_ like plasmids. Following this initial discovery, multiple *mcr*–3 variants were identified with one or more amino acid substitutions [42]. The *mcr*–3 gene variants have now been recorded in both conjugative and non-conjugative plasmids, including incompatibility groups IncA/C_2_-, IncHI_2_-, IncHI_2A_-, IncF_II_/F_IB_-, IncF-, IncP-, IncR-, and IncY-type plasmids [10].

Expression of *mcr*–3 has been shown to impair LPS integrity and decrease the electron density of the LPS in *mcr*–3-positive *E*. *coli* isolates, a characteristic also present in *mcr*–1-positive isolates. Yang and colleagues [42] evaluated the impact of the expression of *mcr*–1 and *mcr*–3 variants (*mcr*–3.1 and *mcr*–3.5) on bacterial fitness by cloning *mcr* variants into pUC–19 and transforming naïve *E. coli* J53. In this study, fitness was measured using in vitro competition assays. They determined that both genes reduced bacterial fitness, but *mcr*–3 variants imposed fewer fitness costs due to compensatory mutations, demonstrating that substituting amino acids A457V and T448I from *mcr*–3.5 had compensatory effects when introduced into *mcr*–3.1. The introduction of A457V and T448I individually resulted in a 45.0% increase in fitness; double substitutions of A457V and T448I resulted in a decrease in fitness. These studies highlight the concept that *mcr*–3 gene variants may be able to encode compensatory mutations that reduce the fitness costs associated with supporting *mcr* genes.

## 6. *mcr*–3 and Related Genes in *Aeromonas*: A Paradigm for the Escape of Resistance Across Genera

As discussed above, organisms intrinsically resistant to polymyxins have the potential to be sources of origin for the *mcr* gene [42]. *Aeromonas* are a species of pathogenic Gram-negative bacillus commonly found in soil, agricultural produce, and fresh or brackish water sources [24,43]. Members of the *Aeromonas* species can act as gene reservoirs, as demonstrated by Khedher and colleagues [44], who performed a mass genome analysis of 64,628 bacterial genomes to determine the origin of the *mcr* gene in water-borne sources. They determined that all 1026 of the *Aeromonas* spp. analysed were positive for potentially transferable *mcr–3* and suggested that *Aeromonas veronii* was the origin of the *mcr*–3 gene. Genes that display high sequence similarity to *mcr*–3 and encode similar levels of polymyxin resistance have been identified as *mcr–3*-related genes and will be referred to as such in this review [45].

The *mcr*–3 gene is widely disseminated as an acquired resistance gene, which provides an alternate explanation for its presence in *Aeromonas* spp. [46,47,48]. However, the evidence that *mcr*–3’s origin is from *Aeromonas* spp. comes from Yin and colleagues [10], who compared 28 *PET–-A* sequences, including *mcr*–3, with human isolates of *K. pneumoniae* from Malaysia, *E. coli* isolates in pigs in Thailand, and *S. enterica* serovar Typhimurium from human stool; these isolates showed 99.8% and 100.0% sequence identity to *mcr*–3. They determined that *mcr*–3 showed 94.1% to 94.8% amino acid identity to proteins found in two *Aeromonas* species: an *Aeromonas hydrophila* isolate from human peritoneal fluid and an *Aeromonas caviae* isolate from lake water in Malaysia. Additionally, *mcr*–3 aligned 75.6% to 84.5% amino acid identity with *mcr*–3-related sequences in eight *Aeromonas* spp., suggesting that *mcr–-*3 in *Enterobacteriaceae* may have originated from *Aeromonas* and that Gram-negative isolates carrying *mcr*–3 or *mcr–-3*-related genes are widely disseminated throughout animals, humans, and the environment [10,45]. Phylogenetic analysis of *mcr*–3 by Shahzad and colleagues [49] further demonstrated that *mcr*–3 evolved from *Aeromonas* spp. Their analysis suggested that *mcr–3* originated from *Aeromonas* with the gradual evolution of *mcr*–3 variants into *E. coli* and *K. pneumoniae,* whereas other *mcr* gene variants evolved from *Klebsiella* spp. and *E. coli*; a total of 43 variants of *mcr*–3 have been discovered as of April 2024 [43,47,49,50,51,52,53,54,55,56].

How did *mcr–3* escape the *Aeromonas* chromosome? It is suggested by Wang and colleagues that *mcr*–3 was first transposed from *Aeromonas* spp. to the *Enterobacteriaceae* family through IS*Kpn40*-mediated transposition [57]. IS*Kpn40* is a member of the mobile IS*3* insertion element family [58]. IS*Kpn40* is found flanking *mcr*–3 and has been shown to mobilise via homologous recombination. Other IS*3* family members, such as IS*911,* can transpose genes via circular intermediates, and it was determined that *mcr*–3 is transposed by a similar mechanism. IS*Kpn40* plays a role in the mechanism behind *mcr*–3’s transposition [59]. However, the roles of other insertion elements flanking *mcr*–3, such as IS*4321* and IS*26*d, and their contributions to *mcr*–3 dissemination across species and genera, have yet to be determined. Notably, IS*Kpn40* is found in *E. coli*, *S. enterica*, *A. caviae*, *A. veronii*, and *K. pneumoniae,* which is suggested to accelerate the dissemination and transmission of *mcr*–3 across these species [59].

**Figure 1:**
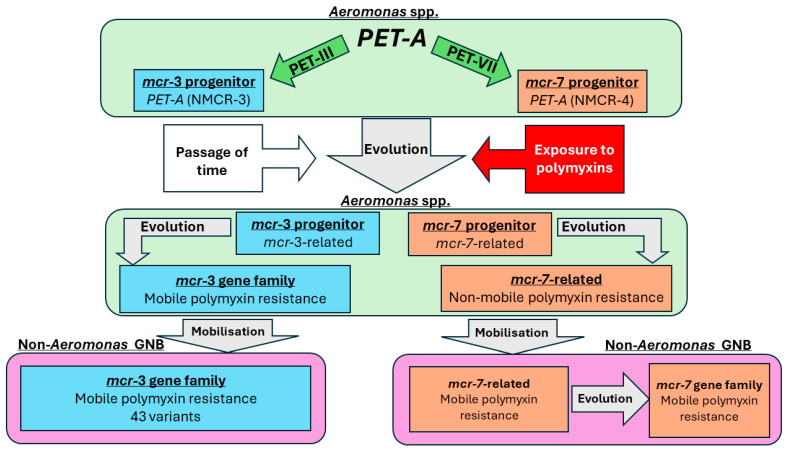
Model of the mobilisation of polymyxin resistance from *PET–A* to *mcr*–3 and 7.

Wang and colleagues’ conclusion that *Aeromonas* is the evolutionary origin point of the *mcr*–3 gene is supported by Guo and colleagues, who performed a mass analysis of 1168 *Aeromonas* genomes [37]. They identified 1825 *PET–A* encoding genes, 334 of which were mobile *mcr* and *mcr-*related genes, and 1491 were non-mobile *PET–A* genes, which they identified as NMCR. Based on their analysis, Guo and colleagues proposed that there are three types of PET–A encoding genes: NMCR/*PET–A*, *mcr*-related, and *mcr* genes. They found that all PET–A encoding genes from *Aeromonas* were divided into three families (PET–III, PET–V, and PET–VII). In the PET–III family, the distribution of clades among NMCR–3, *mcr*–3-related genes, and *mcr*–3 became increasingly smaller layer by layer as the tree progressed. A similar phenomenon was recorded in the PET–VII clade with NMCR–4 and *mcr*–7-related genes but not with *mcr*–7. They concluded that NMCR–3 and NMCR–4 are progenitors of *mcr–3* and *mcr*–7, respectively. The *mcr*-related genes have been suggested as an evolutionary intermediate between the non-mobile NMCR *PET–A* and mobile *mcr* genes [37] (Figure 1). Additionally, Guo and colleagues identified that NMCR–3 progressed from its non-mobile form to a mobile form (*mcr*–3 and *mcr*–3-related genes) within the *Aeromonas* spp., whereas NMCR–4 only underwent partial evolution to *mcr*–7-related genes within *Aeromonas* (Figure 1). Based on their phylogenetic analysis and the fact that *mcr*–7 has not yet been identified in *Aeromonas* and has been recorded in non-*Aeromonas* GNB, they suggested that the complete evolution of the non-mobile NMCR–4 *PET–A* to the mobile *mcr*–7 requires the transfer of *mcr*–7-related genes between genera.

The level of polymyxin resistance imparted by *mcr* gene families varies depending on the organism in which they are found and their point of evolutionary origin. The modification of lipid A in the LPS of bacterial membranes encoded by the *mcr*–3 gene typically mediates lower levels of colistin resistance with minimum inhibitory concentrations (MICs) of less than 8 mg/L^−1^ in members of *Enterobacteriaceae*; however, *mcr* containing *Aeromonas* spp. have been recorded to have polymyxin MICs ranging from 32 to greater than 128 mg/L^−1^ [10,24,60]. It was determined by Ling and colleagues that the acquisition of a single *mcr–3* gene in *Aeromonas salmonicida* increased the MIC against colistin from 1 to 64 mg/L^−1^ [50]. The mechanism behind this high level of colistin resistance imparted by *mcr*–3 is yet to be determined [24]. It has been suggested that the increased level of polymyxin resistance imparted by *mcr*–3 could be the result of synergistic effects between *mcr* and the *arnBCADTEF* operon protein products, which are associated with l–Ara4N modification of lipid A [35]. The synergistic lipid A modifications increased the polymyxin MIC from 1 mg/L^–1^ to 64 mg/L^–1^ in *A. caviae* and *A. salmonicida;* however, the mechanism behind this proposed synergy is yet to be determined.

The *mcr*–3 gene serves as a paradigm for the escape of *mcr* from an intrinsically resistant genus to a susceptible one, as demonstrated by the level of resistance imparted by *mcr*–3 to *Escherichia*, a susceptible genus. The evolutionary mechanism behind the escape of polymyxin resistance from an intrinsically resistant *Aeromonas* into a susceptible organism is yet to be determined [42,43,45].

## 7. Functionality of *mcr*–3 and *mcr*–3-Related Genes in Non-Native Species

While the evidence suggests that *Aeromonas* spp. are the likely evolutionary origin point of the *mcr*–3 gene, the evolutionary relationship between chromosomal *ipet–A* and transferable *mcr–3* is poorly understood [54]. Similarly, the mechanism by which *mcr*–3 encoded PET–A escaped from the chromosome of *Aeromonas* and disseminated into the environment has not been elucidated. The *mcr*–3-related genes are genes that are predicted to catalyse a similar function to *mcr*–3 and have been recorded in *A. hydrophila* and *A. jandaei* chicken isolates in China in 2021 [54]. These genes may play a role in the evolutionary relationship for the escape of polymyxin resistance from chromosomal *ipet–A* to transferable *mcr*–3 (Figure 1) [37,42].

As mentioned in Section 6, *mcr*–3 can impart high levels of polymyxin resistance in intrinsically resistant and naïve organisms. A similar phenomenon has been observed in isolates positive for *mcr*–3-related genes. This was demonstrated by Wang and colleagues, who investigated the prevalence of *mcr*–positive bacteria in 5169 chicken-derived isolates and analysed the genetic environments of their genomes, identifying 14 strains that were positive for *mcr*–3-related genes [54]. An analysis of 16S rRNA gene sequences and whole genome sequences revealed that all isolates positive for *mcr*–3-related genes were from *Aeromonas* spp.; nine were *A. hydrophila* and five were *A. jandaei*. Antimicrobial susceptibility testing revealed that *mcr*–3-related positive isolates had high levels of resistance, with MICs ≥ 128 mg/L. It is unknown whether the high level of polymyxin resistance is the result of synergistic effects between *mcr*–3-related genes, *mcr*–3, and other resistance genes. There is limited current literature on the relationship between *mcr*–3-related genes and polymyxin resistance or the mechanisms behind polymyxin’s escape from the chromosome [22,35,37].

The level of resistance imparted by *mcr*–3-related genes in a naïve organism relative to *mcr*–3 was highlighted in a study by Ling and colleagues, who investigated the functionality of *mcr*–3-related genes in intrinsically resistant *Aeromonas* spp. and naïve *E. coli* [50]. They identified *mcr*–3-related and *mcr*–3.3-related genes in 16 *A. veronii* chicken isolates in China. These gene variants were compared to swine-sourced *E. coli* isolates from NCBI and showed 95.2% and 84.2% nucleotide sequence identity. To determine the functionality of these *mcr* genes, Ling and Yin cloned 1853 bp and 3558 bp sequence fragments, including *mcr*–3.3, *mcr*–3-related fragments, and their respective upstream sequences, into a pUC19 cloning vector that was transformed into a susceptible *E. coli* DH5α. The transformants containing pUC19–*mcr*–3.3 and pUC19–*mcr*–3-related genes had colistin MICs of 2 mg/L and 1 mg/L, respectively, which were four to eight times higher than the MIC of *E. coli* without the presence of *mcr*-related genes and exceeded the clinical break point against polymyxins of 2 mg/L. The investigators then transformed the same constructs into *A. salmonicdia*. The MIC of both transformants increased from 1 to 64 mg/L, a 64-fold increase over the transformant with only pUC19. Stability testing showed that pUC19–*mcr*–3.3-related transformants were stable after 20 generations in the presence and absence of colistin (1 mg/L). The results indicate that *mcr*–3.3-related genes can confer colistin resistance in *Aeromonas* spp. and *E. coli,* and that the functionality of *mcr*–3-related genes remains unclear in non-*Aeromonas* spp. due to the reduced functionality of promoter regions.

## 8. Conclusions

With the emergence and dissemination of multi-drug resistance, it is becoming increasingly important to monitor the use of polymyxin B and colistin as antibiotics of last resort. The *Aeromonas* genus is widely disseminated throughout the environment, so the presence of polymyxin resistance genes in these reservoirs poses a significant risk for the spread of resistance to these antibiotics. Continued surveillance of the genes responsible for imparting this resistance, including *mcr*–3 and *mcr*–3-related genes, is important. Moreover, so is surveillance for emerging resistance phenotypes, as it may be possible for PET–A and IPET–A enzymes from other intrinsically resistant organisms to escape the confines of their genomes.

## Figures and Tables

**Table 1:** First identification of *mcr* gene families by year, location, and organism.

Gene Family	Country	Year	Organism	Sequence Length	References
*mcr*-1	China	2016	*E. coli*	1626 bp	[9]
*mcr*-2	Belgium	2016	*E. coli*	1617 bp	[26]
*mcr*-3 *	China	2017	*E. coli*	1626 bp	[10]
*mcr*-4	Belgium, Spain, and Italy	2017	*E. coli* *Salmonella enterica*	1644 bp	[27]
*mcr*-5	Germany	2017	*Salmonella enterica*	1617 bp	[28]
*mcr*-6	UK	2017	*Moraxella pluranimalium*	1620 bp	[23]
*mcr*-7 *	China	2018	*K. pneumoniae*	ND	[25]
*mcr*-8	China	2018	*K. pneumoniae*	ND	[29]
*mcr*-9	USA	2019	*Salmonella enterica*	ND	[30]
*mcr*-10	China	2022	*Enterobacter roggenkampii*	ND	[31]

* *mcr* gene variants identified in the *Aeromonas* spp.
